# Fatty Acid Oxidation Is Essential for Egg Production by the Parasitic Flatworm *Schistosoma mansoni*

**DOI:** 10.1371/journal.ppat.1002996

**Published:** 2012-10-25

**Authors:** Stanley Ching-Cheng Huang, Tori C. Freitas, Eyal Amiel, Bart Everts, Erika L. Pearce, James B. Lok, Edward J. Pearce

**Affiliations:** 1 Department of Pathology and Immunology, Washington University School of Medicine, St. Louis, Missouri, United States of America; 2 Trudeau Institute, Saranac Lake, New York, United States of America; 3 Department of Pathobiology, University of Pennsylvania School of Veterinary Medicine, Philadelphia, Pennsylvania, United States of America; Rush University Medical Center, United States of America

## Abstract

Schistosomes, parasitic flatworms that cause the neglected tropical disease schistosomiasis, have been considered to have an entirely carbohydrate based metabolism, with glycolysis playing a dominant role in the adult parasites. However, we have discovered a close link between mitochondrial oxygen consumption by female schistosomes and their ability to produce eggs. We show that oxygen consumption rates (OCR) and egg production are significantly diminished by pharmacologic inhibition of carnitine palmitoyl transferase 1 (CPT1), which catalyzes a rate limiting step in fatty acid β-oxidation (FAO) and by genetic loss of function of acyl CoA synthetase, which complexes with CPT1 and activates long chain FA for use in FAO, and of acyl CoA dehydrogenase, which catalyzes the first step in FAO within mitochondria. Declines in OCR and egg production correlate with changes in a network of lipid droplets within cells in a specialized reproductive organ, the vitellarium. Our data point to the importance of regulated lipid stores and FAO for the compartmentalized process of egg production in schistosomes.

## Author Summary

Schistosomes are parasitic worms that are the cause of the Neglected Tropical Disease schistosomiasis. Female schistosomes mated with males produce eggs, which either pass out of the host's body for transmission of the infection, or become trapped in host tissues, where they induce inflammation that contributes to disease symptoms. It has been assumed that egg production is a bioenergetically-demanding process fuelled by glucose metabolism. However, we have discovered that egg production is blocked by inhibition of fatty acid oxidation (FAO), the process through which FA are utilized within mitochondria to fuel the tricarboxylic acid cycle and thereby produce substrates for ATP synthesis through oxidative phosphorylation. Consistent with a role for FAO in egg production, fecund females have extensive fat stores, in the form of lipid droplets, whereas virgin adult females have little or no fat reserves. Moreover, fecund females placed into tissue culture exhaust their fat reserves and cease to be able to produce eggs. Since schistosomes cannot produce their own FA, our data point to the acquisition of FA from the host as a key process necessary for egg production. Our findings point to the importance of regulated lipid stores and FAO for egg production by schistosomes.

## Introduction

Infection with helminth parasites of the genus *Schistosoma* causes chronic and debilitating disease in over 200 million people worldwide [1], [2]. Adult *S. mansoni* worms live within the portal vasculature, producing eggs (200–300/day/female) that are intended to pass into the intestinal lumen for release into the environment to allow transmission of the infection [3]. However, many eggs are carried by the blood flow to the liver, where they become trapped in sinusoids and elicit strong Th2 cell mediated immunopathology, which is the cause of disease manifestations [3]. Since egg production is key for both transmission and pathogenesis, studying reproductive biology in schistosomes could lead to new methods for preventing or treating disease [4].

Adult schistosomes exhibit sexual dimorphism, a trait that is unusual among parasitic trematodes, and display a fascinating codependency: the female resides in a groove (the gynecophoric canal) on the ventral side of the male and is dependent on ongoing physical pairing, but not sperm transfer [5], for proper sexual development [5]–[11]. Virgin adult female schistosomes, from female-only infections, are developmentally stunted compared to fecund females from mixed-sex infections and are unable to lay eggs [11], [12]. Furthermore, egg-laying females that are physically separated from their partners and surgically implanted into a host in the absence of male worms cease egg production and regress reproductively to an immature state. Interestingly, regression is reversible because normal reproductive activity is resumed when separated females are re-paired with males [11], [13], [14]. Regression is largely the result of involution of the vitellarium, a proliferative tissue that occupies the posterior two thirds of the female and produces cells that surround the ovum and provide proteins for eggshell formation and nutrients for the developing embryo [12].

There have been numerous suggestions that male parasites promote female maturation by “providing” nutrients [15]. The fact that starvation in planaria (free living flatworms) can lead to reversible tissue involution [16] is consistent with the possibility that loss of vitelline cells is the end result of nutritional deprivation in female parasites. Glucose is considered to be the key macronutrient required by adult schistosomes to meet their bioenergetics needs [17], [18], but there is a lack of clarity in the literature regarding the relative extent to which Warburg metabolism (the homolactic fermentation of glucose in the presence of oxygen) versus mitochondrial oxidative phosphorylation (OXPHOS) are important in these organisms [17], [19], [20]. Nevertheless, fecund adult females gradually stop ovipositing in vitro even when glucose and oxygen are not limiting [21], and under anaerobic conditions egg production ceases immediately despite the fact that the worms remain viable for extended periods [17]. These findings led us to consider the possibility that worms are able to survive using Warburg metabolism, but require substrates other than glucose for oxidative metabolic pathways critical for egg production. Despite the general view that there is no appreciable lipid catabolism in helminth parasites [18], the genes encoding the enzymes of the β-oxidation pathway, through which fatty acids (FA) are catabolized into the TCA cycle, are conserved in schistosomes [22]. Moreover, greater than 40% of the lipid in adult schistosomes is in the form of triacylglyceride (TG), usually considered an energy store for β-oxidation [23], and FA are able to promote egg production and egg viability in vitro [24]. We therefore decided to ask whether adult female schistosomes use FA oxidation (FAO) for egg production.

## Results/Discussion

### Fecund female schistosomes use OXPHOS

**Figure 1:**
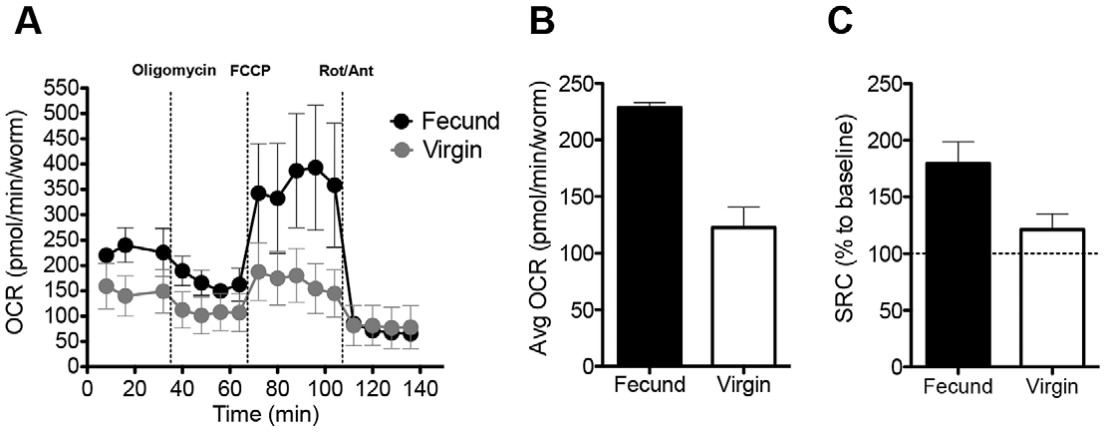
Fecund female schistosomes have high mitochondrial OCR. **A.** OCR of Fecund female parasites recovered from mixed sex infections, and Virgin adult females recovered from single sex infections were measured in real time by extracellular flux analysis, at basal (immediately ex-vivo) and following the addition of oligimycin, FCCP and rotenone (Rot) and antimycin A (Ant) at the times indicated. **B.** Average basal OCR readings of Fecund and Virgin females over the first 30 minutes ex-vivo. **C.** Spare respiratory capacity of Fecund and Virgin females, calculated as shown in Fig. S1. Data are means plus SEM of readings from 4–5 individual female worms per experiment. Data are representative of at least 3 individual experiments. See also Fig. S1.

The β-oxidation pathway allows FA to be used as fuel for the TCA cycle, which in turn generates substrates for the electron transport chain to make ATP via OXPHOS. To examine whether this process occurs in adult female schistosomes, we used extracellular flux analysis to compare mitochondrial oxygen consumption rates (OCR, [25]) in individual fecund and virgin female schistosomes immediately ex vivo (Fig. S1). OCR in female schistosomes declined in the presence of oligomycin, and antimycin-A plus rotenone (Fig. 1A), indicating that it is largely a function of mitochondrial OXPHOS (Fig. S1). Baseline OCR (Fig. 1A,B), and mitochondrial spare respiratory capacity (SRC, Fig. 1A,C) [26], were significantly higher in fecund vs. virgin females (P<0.01). SRC is the difference between OCR at basal state and after addition of FCCP (Fig. S1), and reflects the extra mitochondrial capacity available to produce energy under conditions of increased work or stress and is an important determinant of long-term cellular survival and function [26], [27]. Since the sizes of fecund and virgin adult females differ [21], the SRC measurement also provides an internally controlled indication that there are significant qualitative differences in mitochondrial respiration between fecund and virgin worms.

### OXPHOS is essential for egg production

**Figure 2:**
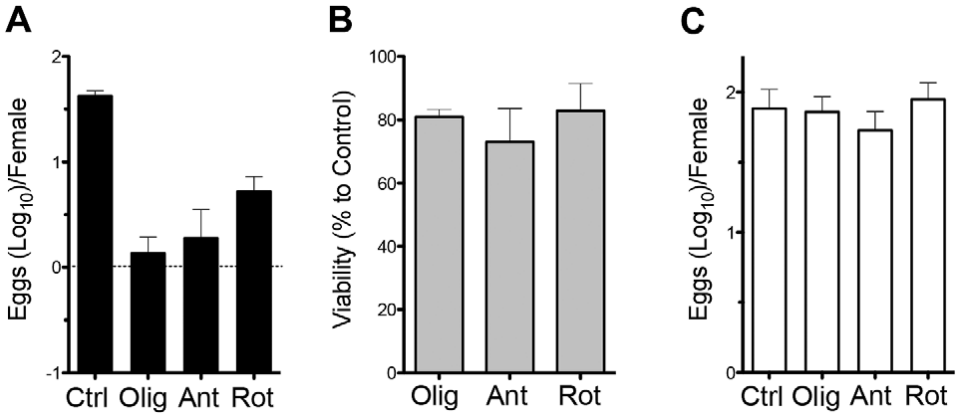
OXPHOS is necessary for egg production. **A.** Eggs produced per fecund female during the first 24 h ex vivo, in the absence (Ctrl) or presence of oligomycin (Olig), antimycin A (Ant) or rotenone (Rot). **B.** Survival of females, compared to untreated cultured worms, during the same period and conditionS as described in A. **C.** Egg production between 24 h and 48 h in vitro following the washing out of inhibitors that were present during the first 24 h ex vivo. Data are means plus SEM of readings from 10 individual female worms per experiment. Data are representative of at least 3 individual experiments.

Previous work showed that female schistosomes require oxygen to produce eggs [17]. To assess whether these findings reflect a dependence on OXPHOS, we cultured fecund female worms for 24 h in oligomycin, antimycin A or rotenone, all of which inhibit mitochondrial OCR (Fig. 1), and measured egg production and worm viability; these inhibitors had a significant (p<0.01 in each case) negative effect on egg production (Fig. 2A), but little adverse effect on worm viability over this time period (Fig. 2B). Moreover, when the inhibitors were washed out after 24 h, egg production resumed at normal levels over the ensuing 24 h (Fig. 2C). These data indicate that female worms can survive independently of mitochondrial respiration, but absolutely require this process in order to produce eggs.

### Cpt1 activity regulates oxygen consumption and egg production

**Figure 3:**
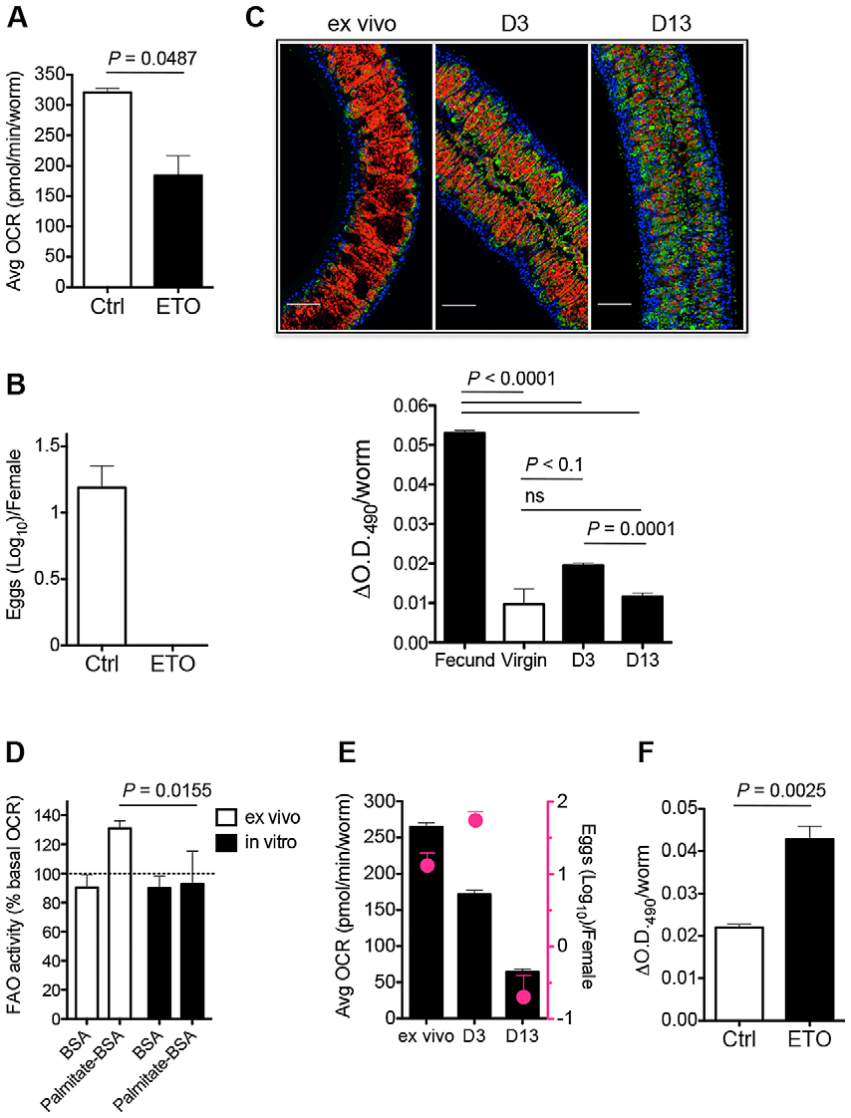
Schistosomes use FA from lipid droplets for FAO to produce eggs. **A.** Average basal OCR of fecund females incubated without (Ctrl) or with etomoxir (ETO) over the first 30 min ex vivo. See also Fig. S2. **B.** Numbers of eggs produced in 24 h per female parasite in the absence or presence of etomoxir. **C** Oil-Red-O stained fecund females immediately ex vivo or at day 3 or day 13 of culture (red = Oil Red O; blue = Hoescht; green = phalloidin) and quantitation of Oil-Red-O staining of females, as indicated. Images are optical sections through longitudinal axes. Scale bar = 50 µm. **D.** Palmitate induced mitochondrial FAO (% basal OCR) of fecund females ex vivo and after 13 days in culture. **E.** Average basal OCR of fecund females ex vivo and after 3 or 13 days in culture (black bars) and numbers of eggs produced within the 24 h period immediately ex vivo or in the 24 h period prior to day 3 or day 13 of culture (pink circles). **F.** Quantitation of Oil-Red-O staining of fecund females cultured without or with etomoxir for 24 h. Data are means plus SEM of readings from 5–6 individual female worms per experiment. Data are representative of at least 3 individual experiments. ns = not significant.

Carnitine palmitoyl transferase 1 (Cpt1) catalyzes the initial rate limiting step in FAO in which FA are transferred from the cytosol into the mitochondria [28]. To determine whether OXPHOS depends on FAO we incubated fecund female worms with the Cpt1 inhibitor etomoxir [29], [30] immediately ex vivo and measured OCR. We found that etomoxir caused a significant decline in basal OCR (Fig. 3A; S2), without affecting basal extracellular acidification, an indicator of glycolysis (data not shown). Since OXPHOS is essential for egg production (Fig. 2), we reasoned that if FAO is a significant source of substrates for the TCA cycle and therefore for OXPHOS, then inhibition of FAO should have a deleterious effect on egg output. To examine this we recovered fecund female worms from infected mice and measured the effect of etomoxir on egg production over 24 h in culture. Under these conditions, etomoxir completely suppressed egg production (Fig. 3B), although worms remained viable. These data implicate FAO in egg production by female schistosomes.

### Fecund female schistosomes have extensive lipid reserves

The understanding of how FA are utilized by cells is evolving rapidly. The current view is that FA are converted into TG and stored in cytoplasmic lipid droplets, from which they are released in a regulated fashion by lipolysis [31] to be used as energy substrates in FAO, or as ligands for nuclear receptors. It has been reported that schistosomes possess considerable TG stores when recovered from mice [23], but the function and location of these stores remains enigmatic [22]. To examine this we stained female worms immediately ex vivo with Oil-Red-O, which binds to neutral TG and was recently authenticated as a true lipid stain in the free-living helminth *Caenorhabditis elegans* [32]. The results were striking, revealing that fecund female parasites possess an extensive lipid droplet network. This network was evident microscopically, and by measuring extracted dye spectrophotometrically (Fig. 3C). In contrast, virgin females had significantly lower lipid reserves (Fig. 3C). Moreover, the intensity of Oil-Red-O staining declined markedly over time as fecund worms were maintained in tissue culture for 3 or 13 days (Fig. 3C). Previous reports have commented on the presence of lipid droplets within mature (Stage 4) vitelline cells [13]. Although we do note have proof that all of the droplets that we have visualized using Oil-Red-O staining and confocal microscopy are within the vitellarium, their location is anatomically consistent with the majority of them being associated with this organ. The lack of Oil-Red-O staining in virgin worms, and in fecund females after culture, is consistent with the failure of the vitellarial lineage to produce Stage 4 cells under these conditions [10], [13].

The decline in lipid reserves in vitro is of interest since it occurs with a similar kinetic to the decline in egg production by cultured worms [21]. We reasoned that this could reflect a causal link between lipid droplet exhaustion and the cessation of FAO under these conditions. To explore this, we used real time flux analysis to measure FAO activity and mitochondrial OCR in fecund females immediately ex vivo and in vitro. We found that cumulative levels of palmitate oxidation and basal OCR declined significantly in vitro (Fig. 3D and 3E), and that as anticipated this was paralleled, between days 3 and 13, by a significant decline in egg production (Fig. 3F). To formally examine whether there is a link between FAO and lipid droplet depletion in vitro, we recovered fecund females from infected hosts and cultured them with etomoxir for 24 h and used Oil-Red-O staining to quantify lipid droplets. We found that etomoxir significantly inhibited depletion of lipid reserves in these worms (Fig. 3G).

### FA catabolism is essential for egg production

**Figure 4:**
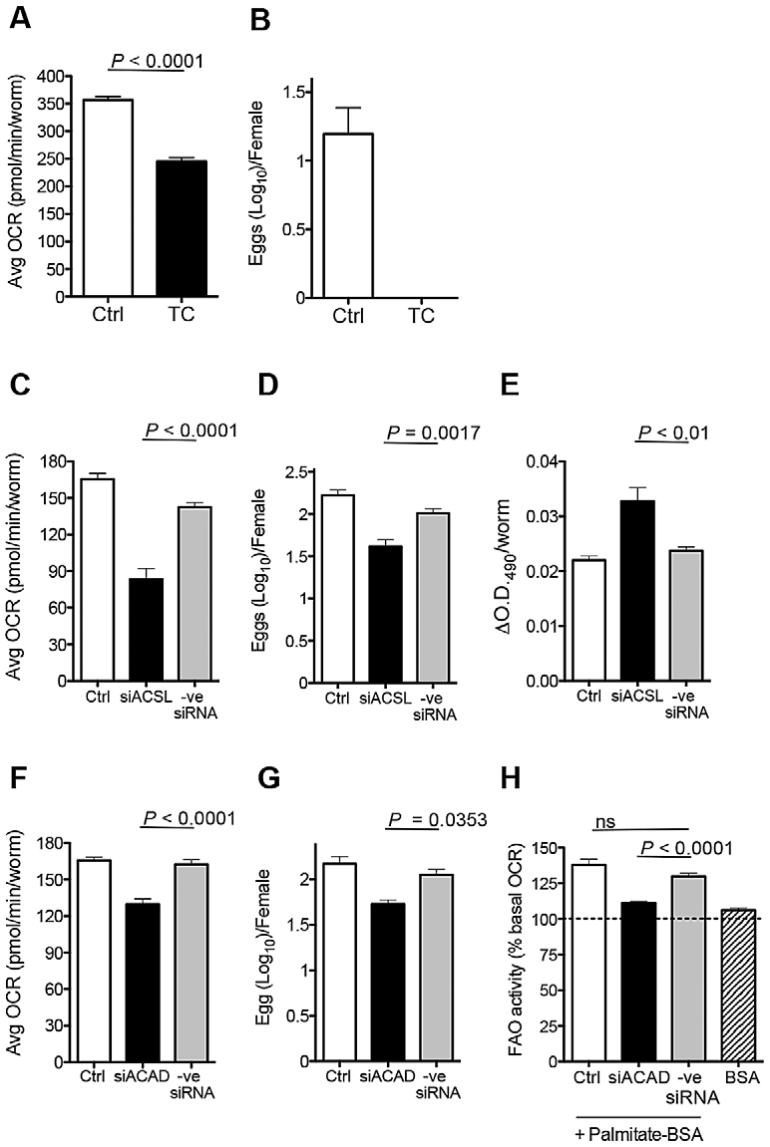
Loss of ACSL and ACAD function inhibits egg production. **A.** Average basal OCR of fecund females incubated without (Ctrl) or with Triascin C (TC) over the first 30 min ex vivo. **B.** Numbers of eggs produced in 24 h per female parasite in the absence or presence of Triascin C. Average basal OCR (**C & F**), numbers of eggs produced in 72 h per female (**D & G**) and quantitation of Oil-Red-O staining (**E**) and measurement of FAO activity (**H**) in control fecund females, and in fecund females electroporated with SmASCL-siRNA (siASCL) or SmACAD-siRNA (siACAD), or with control siRNA (-ve siRNA). Data are means plus SEM of readings from 5–6 individual female worms per experiment. Data are representative of at least 2 individual experiments. See also Fig. S2.

FA liberated from lipid droplets by lipolysis are activated and shuttled into mitochondria for FAO by acyl-CoA synthetase (ACSL) [33], [34]. We reasoned that if FA are essential for OXPHOS and egg production, then loss of function of ACSL should affect both of these parameters by preventing the use of FA resulting from lipolysis. We examined this using chemical inhibitors and RNAi. First, we tested the effect of the fungal metabolite Triacsin C, which is a potent inhibitor of most mammalian ACSLs [35], [36]. We recovered fecund female parasites from their hosts and immediately assessed the effect of Triacsin C on basal OCR and egg production. We found that Triacsin C inhibited OCR (Fig. 4A) and blocked egg production entirely (Fig. 4B). We used RNAi to substantiate the importance of ACSL in OXPHOS and egg production. Immediately after explantation, and prior to assessing OCR and egg production, fecund females were electroporated with siRNAs against SmACSL, or control siRNAs, [37]. Using this approach, SmACSL expression was significantly attenuated within 72 h (Fig. S2A). Concomitant with reduced expression of SmACSL there were significant declines in OCR (Fig. 4C) and egg production (Fig. 4D). Moreover, SmACSL-siRNA resulted in greater retention of lipid reserves over 3 days in culture (Fig. 4E), which was also apparent to some extent in Triascin-C treated worms (Fig. S2B).

The initial step in mitochondrial β-oxidation is catalyzed by acyl-CoA dehydrogenase (ACAD). We targeted schistosome ACAD using siRNAs; this approach resulted in reduced ACAD mRNA, (Fig. S3C), decreased mitochondrial OCR (Fig. 4F), and decreased egg production (Fig. 4G). Furthermore, the stimulation of FAO by added palmitate was significantly impaired by this siRNA treatment (Fig. 4H).

Taken together, these data support a role for the mobilization of lipid droplet reserves for FAO in female schistosomes, and the use of this pathway to support egg production.

Schistosomes cannot synthesize their own FA [22], but they can take up lipids and convert them into TG [23], [38]. Therefore we propose that in vivo, TG in lipid droplets are continuously catabolized for FAO and replenished through the uptake of FA from the environment. We hypothesize that FAO is essential for the differentiation and/or survival of Stage 4 vitelline cells. In this model, the reduced OCR and SRC of virgin vs. fecund females are due to the absence of mature vitellocytes that normally are committed to FAO and OXPHOS. Our data indicate that, in vitro, lipid stores are used but not replenished, thereby accounting for the loss of Oil-Red-O staining and declines in OCR as TG reserves are depleted in cultured parasites. Our data fit with the view that reproductive maturation and regression are closely linked to nutritional status in female schistosomes [15], and point to FA as a key nutritional requirement for this process.

How male parasites help females to acquire FA remains to be determined. Schistosomes eat blood, and it has been proposed that male worms physically assist females in this process. However, we have been unable to show any positive effect in the assays described herein of adding red blood cells to cultures of schistosomes, regardless of whether males are present or not (data not shown). Since glucose is an essential nutrient for schistosomes (Krautz-Peterson et al. 2010, and data not shown), it is possible that virgin females are subsisting largely on glucose absorbed directly from the blood through tegumental surface transporters [39], [40]. A plausible explanation for the observation that females cease egg production in vitro, even when male worms are present, is that certain FA present in vivo are missing in the media that have been routinely used to culture schistosomes. Possibilities include short chain FA, which are present in high concentrations in portal blood, and which interestingly are depleted in plasma samples from schistosome-infected mice [41], [42], and stearic acid, which when complexed with bovine serum albumin is able to replace fetal calf serum in a defined medium that is able to support short term egg production by cultured schistosomes [24].

It has been assumed that FAO does not occur in schistosomes, and that glucose is the key substrate for energy generation. However, the data presented here indicate that schistosomes use FAO specifically for the compartmentalized process of egg production. A role for FAO in schistosome egg production is consistent with the important roles of FA in reproduction in insects and mammals [43], [44]. It will be important to identify the FA that support egg production and to understand the specific mechanism by which male schistosomes assist females in acquiring these nutrients. Unraveling the metabolic requirements for reproduction in schistosomes may enable development of enhanced tissue culture systems that will support continuous egg production in vitro. This, in turn, would greatly facilitate the application of emerging tools for transgenesis in these important parasites [45]. Moreover, it is conceivable that a greater understanding of the metabolic processes that support schistosome egg production may offer new opportunities to simultaneously prevent transmission and disease development.

## Materials and Methods

### Ethics statement

This study was carried out in strict accordance with the recommendations in the Guide for the Care and Use of Laboratory Animals of the National Institutes of Health. The protocol was approved by the Institutional Animal Care and Use Committee of Washington University School of Medicine (Animal Welfare Assurance Number: A-3381-01).

### Parasites and animals

Seven to eight wk old adult *Schistosoma mansoni* (NMRI strain) were recovered from infected C57BL/6 female mice (Jackson Laboratory). Parasites were cultured in RPMI containing 10% fetal calf serum (FCS) (both from GIBCO), 2% antibiotic/antimycotic, 1% HEPES, 10 mM glucose, 2 mM L-glutamine and 1 mM sodium pyruvate (all from Sigma) at 37°C in 95%air/5%CO_2_. Medium was replaced every 3 days. Eggs produced every 24 h were counted using a microscope.

### Metabolism assays

Real-time measurements of OCR and extracellular acidification were made using an XF-24 Extracellular Flux Analyzer (Seahorse Bioscience). Worms were plated in XF-24 Islet Capture Microplates (one worm per well) and analyzed in non-buffered RPMI 1640, 25 mM glucose, 10% FCS, 100 U/mL penicillin/streptomycin, 2 mM L-glutamine and 1 mM sodium pyruvate under basal conditions or in the presence of oligomycin (3 µM), fluoro-carbonyl cyanide phenylhydrazone (FCCP, 4.5 µM), rotenone (0.3 µM), antimycin A (3 µM), etomoxir (200 µM) (all from Sigma) or Triacsin C (10 µM, Enzo Life Sciences). For FAO assay, real-time oxidation rates of palmitate in worms was assessed by extracellular flux analysis as described above. Basal OCR rates were measured prior to 2 h treatment with palmitate (200 µM) with fatty acid free bovine serum albumin (BSA), or with fatty acid free BSA (0.17 µM) alone (Seahorse Bioscience).

### Treatment of adult *S. mansoni* with small interfering RNA (siRNA)

siRNA targeting acyl-CoA synthetase (ACSL; GI: 256090263 and GI: 238666949) and acyl-CoA dehydrogenase (ACAD; GI: 353231171 and GI: 256070604) were designed and synthesized by Ambion, Applied Biosystems (*Silencer* Select Custom Designed siRNA; http://www5.appliedbiosystems.com/tools/sirna/). siACSL: sense- GCAUACAGAUGGAAGUUUAtt; antisense-UAAACUUCCAUCUGUAUGCat. siACAD: sense-GGAAUCAAAUGAUAUCUUAtt; antisense-UAAGAUAUCAUUUGAUUCCat. Silencer Negative control siRNA#1, which is not matched to any sequence in the parasite genome, was also provided by the manufacturer and used as a control. siRNA (10 µM) was delivered by electroporation [46].

### Oil-Red-O staining and quantification

Parasites were fixed in 4% paraformaldehyde (Electron Microscopy Sciences) diluted in PBSTx (PBS, 0.3% Triton X-100) for 1 h, [47], dehydrated in 60% isopropanol for 15 min, stained with Oil-Red-O (Sigma) overnight [48], washed in PBSTx 4 times and stained with phallotoxin-Alex Fluor 488 (Invitrogen) and Hoechst at 4°C for 1 h prior to imaging using a Leica SP5 LSCM confocal microscope and a PL APO CS 20× NA = 0.70 objective in the format of 2048×2048. To quantify Oil-Red-O staining, dye was eluted in 100% isopropanol for 30 min and absorbance of the eluate vs. 100% isopropanol at 490 nm was measured [48].

### Statistical analyses

The significance of observed differences was assessed using Student's t-test.

## Supporting Information

Figure S1 **Fundamental parameters of mitochondrial function.** Related to Fig. 1. The XF-24 Extracellular Flux Analyzer, (Seahorse) was used to measure OCR as a basal rate, and after the addition of Oligomycin (an inhibitor of the mitochondrial ATP synthase, FCCP (to uncouple ATP synthesis from the electron transport chain, ETC), or Antimycin A and Rotenone (to block complex I and III of the ETC, respectively), as indicated. Resulting changes in OCR indicate the amount of oxygen consumed for mitochondrial ATP production, the maximal mitochondrial respiration rate when proton flux is uncoupled from ATP synthesis, and finally the amount of oxygen that is consumed by non-mitochondrial processes when the ETC is inhibited. The SRC (spare respiratory capacity) is the difference between maximal and basal OCRs.
(DOCX)

Figure S2 **Dose response to etomoxir.** Average basal OCR of fecund females incubated without (0) or with etomoxir (ETO) at different concentrations, over the first 30 min ex vivo. Data are means plus SEM of readings from 5–6 individual female worms per experiment.
(DOCX)

Figure S3 **RNAi-mediated knockdown of, and targeted inhibition of, SmACSL and SmACAD. Related to** **Fig. 4** **.** Treatment of fecund parasites immediately ex-vivo with siRNA specific for SmACSL (**A**) or SmACAD (**C**) led to a 50%–60% reduction in encoding mRNAs after 72 h. Data points are means plus SEM of readings from 5–6 individual female worms per experiment. For real time RT-PCR, RNA was extracted using RNeasy (Qiagen), contaminating genomic DNA was removed using Turbo DNA-free endonuclease (Ambion) and cDNA was synthesized using SuperScript II reverse transcriptase (Invitrogen), and oligo dT. RT-minus controls were performed to confirm absence of genomic DNA (data not shown). SmACSL transcripts were quantified relative to α-tubulin using Applied Biosystems' 7500 real-time PCR system and SYBR green PCR Master Mix (Applied Biosystems), and the 2^−ΔΔCt^ method. Dissociation curves were generated for each real-time RT-PCR to verify the amplification of only one product. SmACSL primers were: forward 5′-TATGCCTCTGCCCAACTCTC-3′ and reverse 5′-CACGTACGGGAAGTGCTAAA-3′. SmACAD primers were: forward 5′-GCTGTCACACCACCTTGTCC-3′ and reverse 5′-TCCAGATTGACTTGGCCTCT-3′. α-Tubulin (GI: 8355916) primers were: forward 5′-TAGAGCGTCCAACCTACACAA-3′ and reverse 5′-GGAAGTGGATACGAGGATAAGG-3′. **B.** Quantitation of Oil-Red-O staining of fecund females cultured without (Ctrl) or with Triacsin C (TC) for 24 h.
(DOCX)
