# Supplementary figures and images for: Fatty Acid Oxidation Is Essential for Egg Production by the Parasitic Flatworm Schistosoma mansoni
Source: PLoS Pathog. 2012 Oct 25;8(10):e1002996. doi: 10.1371/journal.ppat.1002996 (PMC3486914; doi:10.1371/journal.ppat.1002996)

**
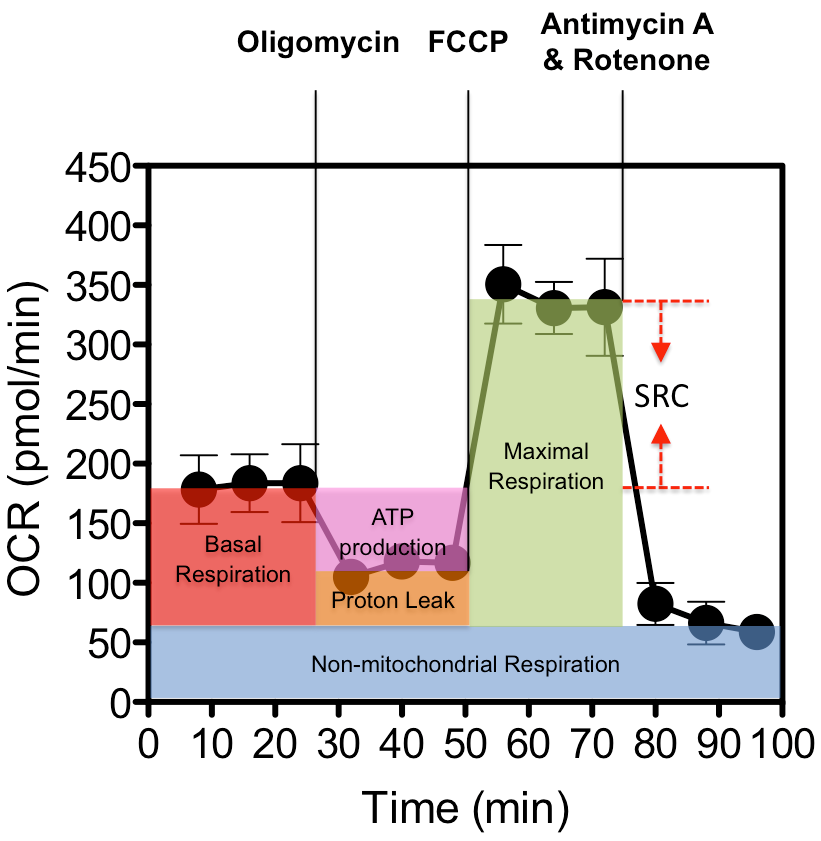
**

Supplement: Figure S1 — Fundamental parameters of mitochondrial function. Related to Fig. 1. The XF-24 Extracellular Flux Analyzer, (Seahorse) was used to measure OCR as a basal rate, and after the addition of Oligomycin (an inhibitor of the mitochondrial ATP synthase, FCCP (to uncouple ATP synthesis from the electron transport chain, ETC), or Antimycin A and Rotenone (to block complex I and III of the ETC, respectively), as indicated. Resulting changes in OCR indicate the amount of oxygen consumed for mitochondrial ATP production, the maximal mitochondrial respiration rate when proton flux is uncoupled from ATP synthesis, and finally the amount of oxygen that is consumed by non-mitochondrial processes when the ETC is inhibited. The SRC (spare respiratory capacity) is the difference between maximal and basal OCRs. (DOCX) [file ppat.1002996.s001.docx]
