# Supplementary material for: Fatty Acid Oxidation Is Essential for Egg Production by the Parasitic Flatworm Schistosoma mansoni
Source: PLoS Pathog. 2012 Oct 25;8(10):e1002996. doi: 10.1371/journal.ppat.1002996 (PMC3486914; doi:10.1371/journal.ppat.1002996)
Supplement: Figure S3 — RNAi-mediated knockdown of, and targeted inhibition of, SmACSL and SmACAD. Related to Fig. 4 . Treatment of fecund parasites immediately ex-vivo with siRNA specific for SmACSL (A) or SmACAD (C) led to a 50%–60% reduction in encoding mRNAs after 72 h. Data points are means plus SEM of readings from 5–6 individual female worms per experiment. For real time RT-PCR, RNA was extracted using RNeasy (Qiagen), contaminating genomic DNA was removed using Turbo DNA-free endonuclease (Ambion) and cDNA was synthesized using SuperScript II reverse transcriptase (Invitrogen), and oligo dT. RT-minus controls were performed to confirm absence of genomic DNA (data not shown). SmACSL transcripts were quantified relative to α-tubulin using Applied Biosystems' 7500 real-time PCR system and SYBR green PCR Master Mix (Applied Biosystems), and the 2−ΔΔCt method. Dissociation curves were generated for each real-time RT-PCR to verify the amplification of only one product. SmACSL primers were: forward 5′-TATGCCTCTGCCCAACTCTC-3′ and reverse 5′-CACGTACGGGAAGTGCTAAA-3′. SmACAD primers were: forward 5′-GCTGTCACACCACCTTGTCC-3′ and reverse 5′-TCCAGATTGACTTGGCCTCT-3′. α-Tubulin (GI: 8355916) primers were: forward 5′-TAGAGCGTCCAACCTACACAA-3′ and reverse 5′-GGAAGTGGATACGAGGATAAGG-3′. B. Quantitation of Oil-Red-O staining of fecund females cultured without (Ctrl) or with Triacsin C (TC) for 24 h. (DOCX) [file ppat.1002996.s003.docx]

*P* = 0.0074

**B**

*P* = 0.0086

**A**

**C**

*P* = 0.0007
