# Supplementary material for: Fatty Acid Oxidation Is Essential for Egg Production by the Parasitic Flatworm Schistosoma mansoni
Source: PLoS Pathog. 2012 Oct 25;8(10):e1002996. doi: 10.1371/journal.ppat.1002996 (PMC3486914; doi:10.1371/journal.ppat.1002996)
Supplement: Supplementary file 3 [file ppat.1002996.s002.docx]

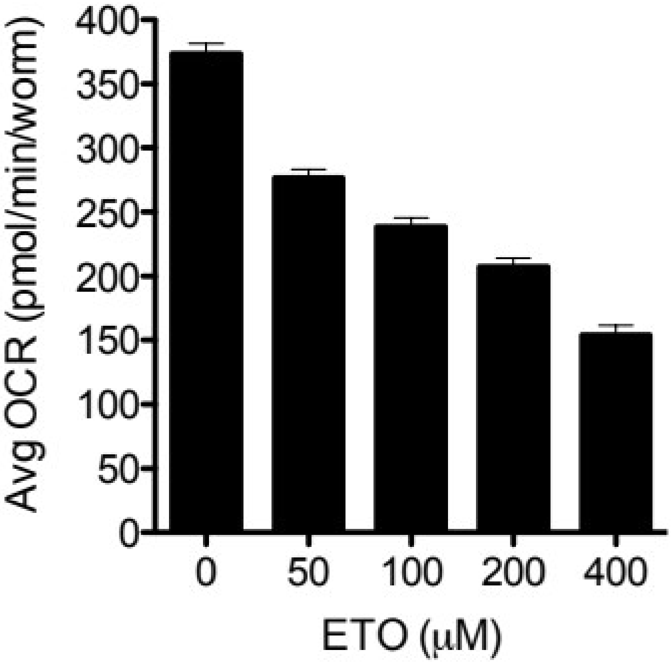

Avg OCR (pmol/min/worm)
400
350
300
250
200
150
100
50
0
0
50
100
200
400
ETO (μM)
